# Supplementary material for: Preparation of Duplex Sequencing Libraries for Archival Paraffin-Embedded Tissue Samples Using Single-Strand-Specific Nuclease P1
Source: Int J Mol Sci. 2022 Apr 21;23(9):4586. doi: 10.3390/ijms23094586 (PMC9105346; doi:10.3390/ijms23094586)
Supplement: Supplementary file 1 [file ijms-23-04586-s001.zip › ijms-1676235-supplementary.pdf]

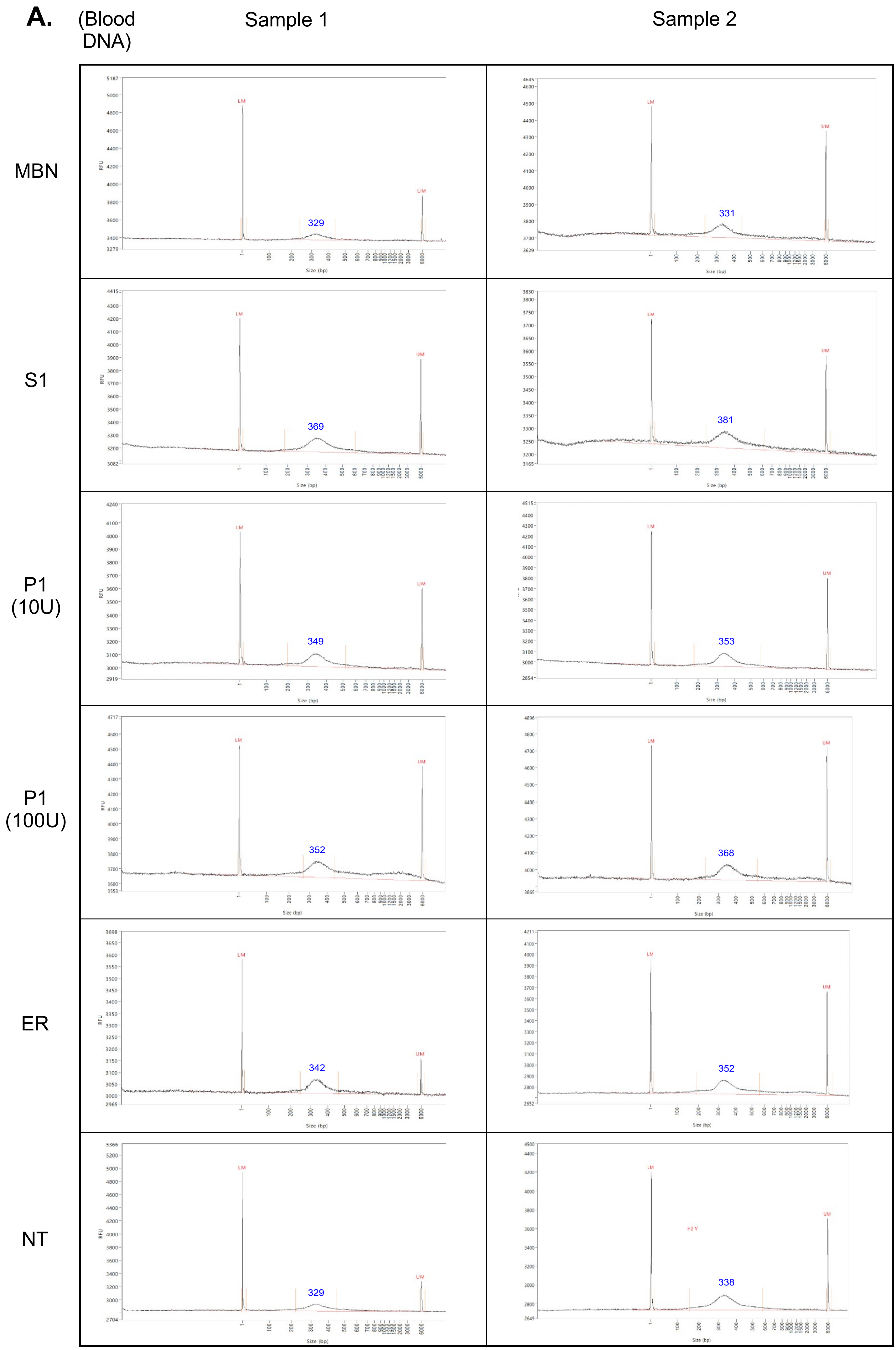
A.
(Blood DNA)
Sample 1
Sample 2
MBN
S1
P1 (10U)
P1 (100U)
ER
NT
329
331
369
381
349
353
352
368
342
352
329
338
LM
UM
RFU
Size (bp)

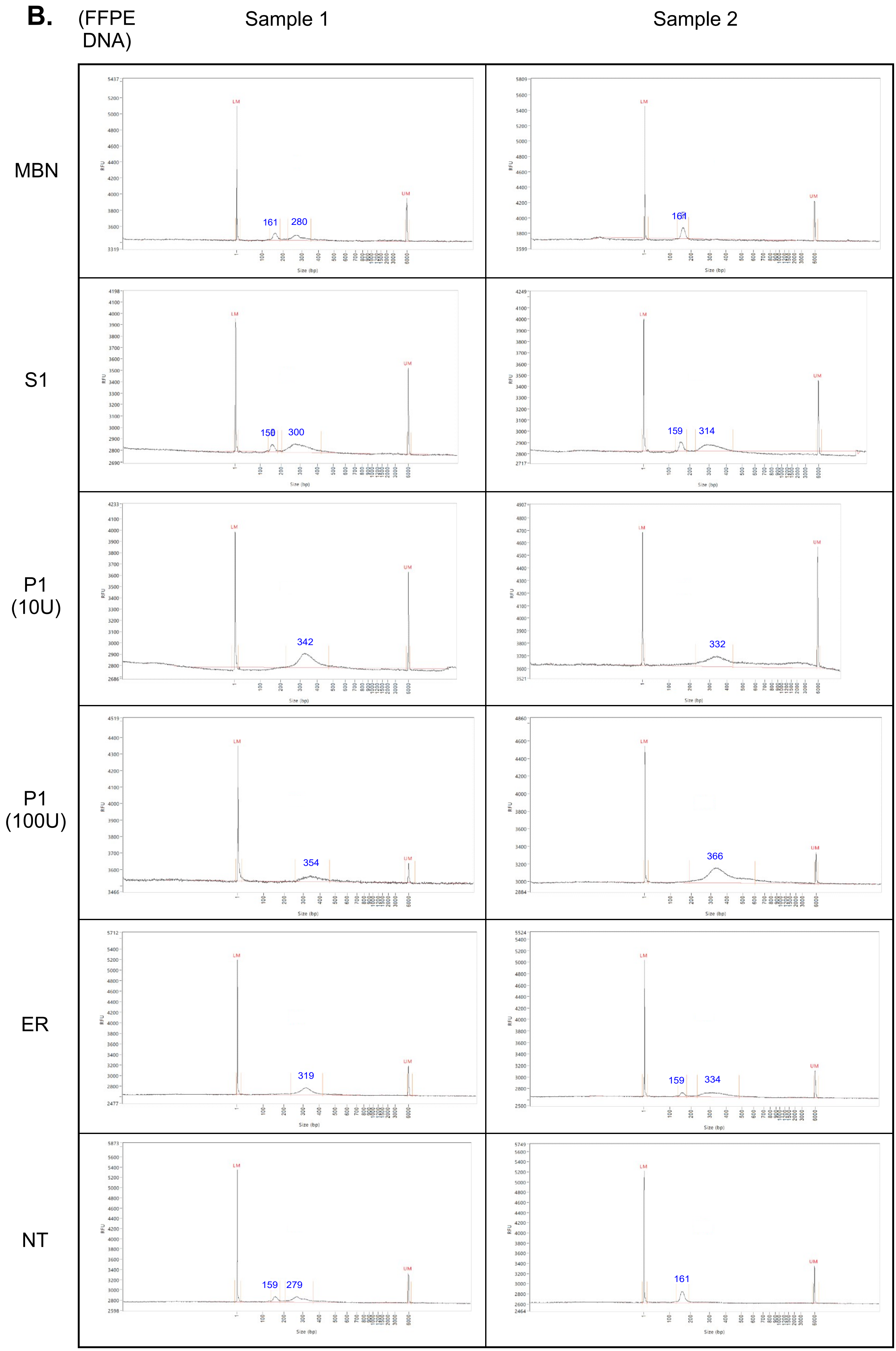
B.
(FFPE DNA)
Sample 1
Sample 2
MBN
S1
P1 (10U)
P1 (100U)
ER
NT
161
280
161
300
159
314
342
332
354
366
319
159
334
159
279
161
LM
UM
RFU
Size (bp)

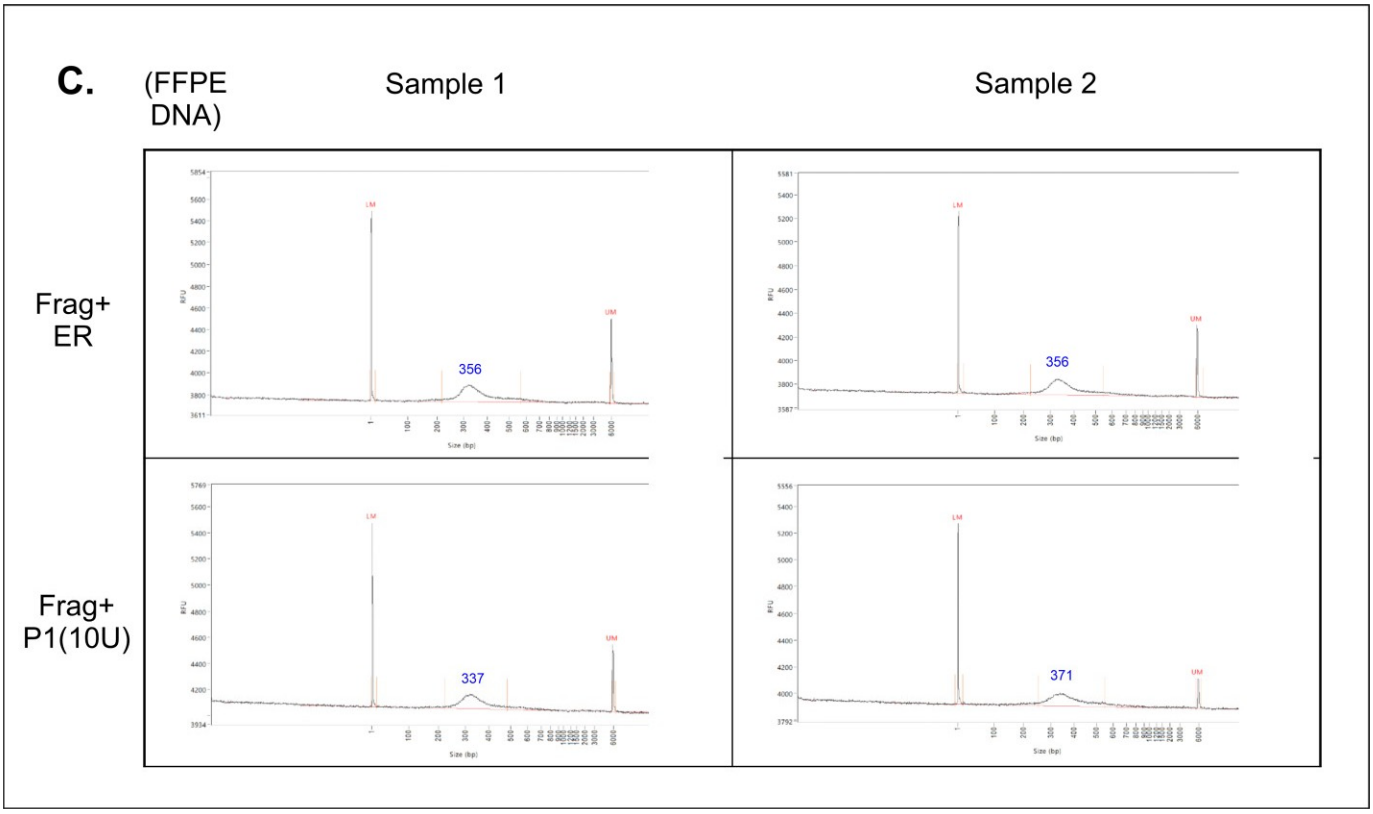

Figure S1. Fragment analysis results. Libraries, prepared as indicated below, were subjected to PCR amplification and their sizes were assessed using 5200 Fragment Analyzer System (Agilent Technologies, USA). (**A**) Separate aliquots of the blood DNA samples 1 and 2 were enzymatically fragmented, then the ends of the resulting DNA fragments were blunted either with one of the single-strand specific nucleases (mung bean nuclease, MBN; S1 nuclease, S1; nuclease P1, P1), or by using a standard polymerase-based approach (ER). One aliquot of each DNA sample was left untreated (NT). Adapters were ligated as described in the Materials and Methods section. (**B**) FFPE-derived DNA samples 1 and 2 were subjected directly to the standard end repair or nuclease treatment without prior fragmentation. (**C**) Two aliquots of each FFPE-derived DNA sample were subjected to enzymatic fragmentation (Frag) before proceeding with standard DNA ends repair (Frag+ER) or nuclease P1 treatment (Frag+P1(10U)). The average fragment size is shown for each library; narrow peaks with sizes of 150-161 bp represent adapter dimers.

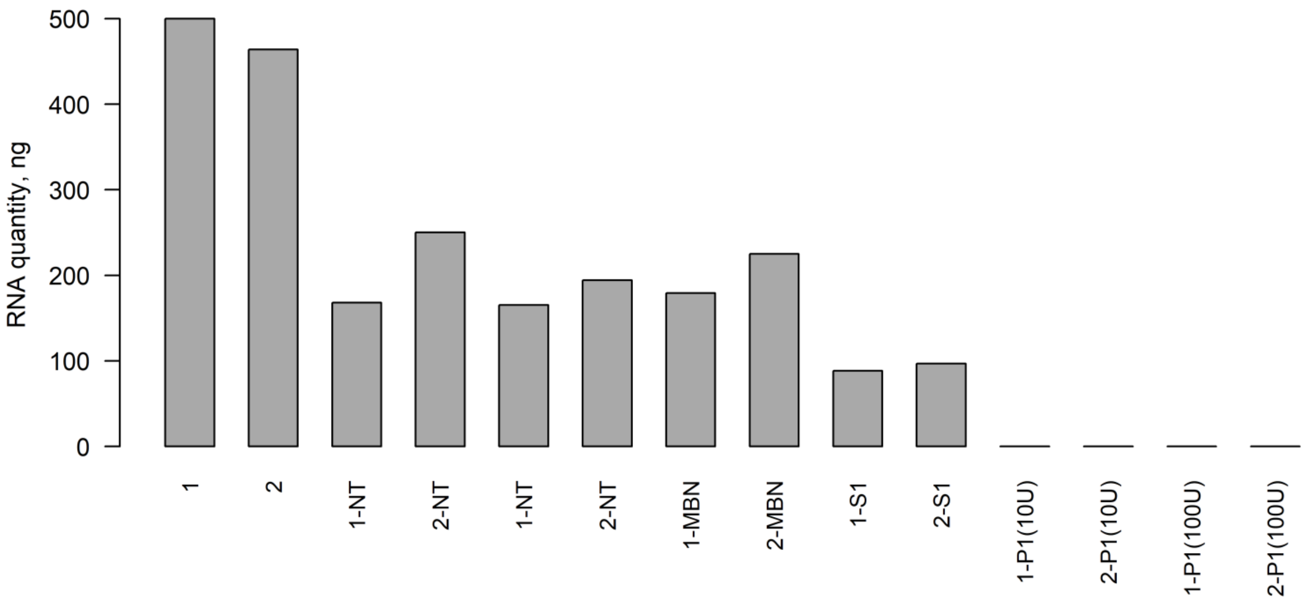

Figure S2. Amount of RNA in DNA preparations treated with single-strand specific nucleases. 1,2 – RNA amount in samples 1 and 2 before any treatment. Each sample aliquot contained 1000 ng of total nucleic acids (RNA+DNA). **NT** – no treatment; the decrease in RNA levels in these cases was attributed to the column purification only. One of the NT aliquots from each sample was further subjected to the standard end repair process. Other aliquots were treated with one of the following enzymes before column purification: **MBN** – mung bean nuclease; **S1** – S1 nuclease; **P1(10U)** and **P1(100U)** – two different concentrations of nuclease P1.

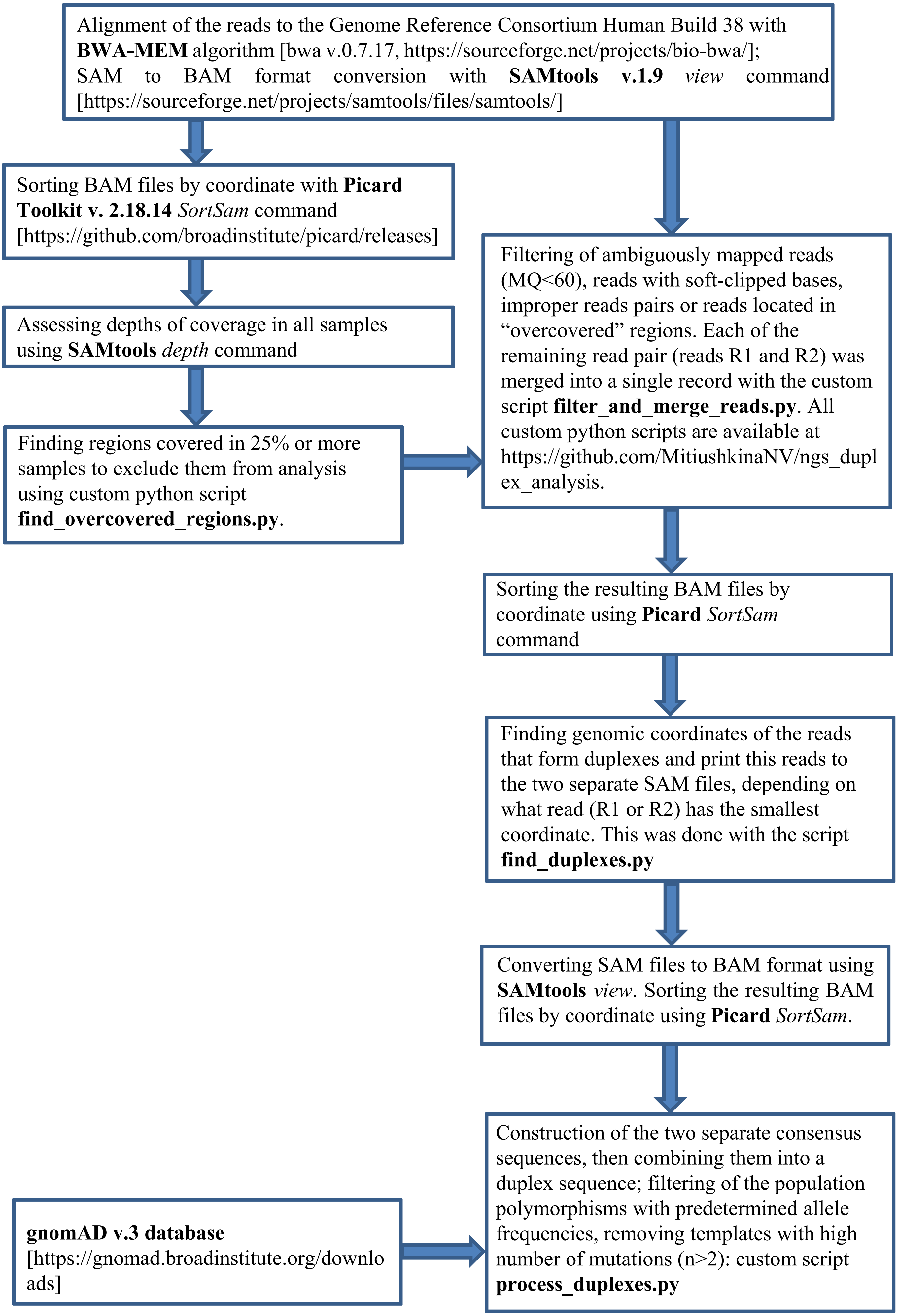

Figure S3. Bioinformatic analysis pipeline.

Table S1. Main results of NGS analysis of 23 BotSeqS libraries.

Sample	Tissue	Total number of reads	Reads passed filters	Median template length	Number of unique templates	Number of duplexes	Total length of duplex sequences	True mutations (present on both strands)	Number of templates with true mutations	SNP with population frequency >1%*	Number of errors corrected by duplex sequencing
MSI#1	Tumor	1370414	1021326	167	13907	2829	356570	39	37	345	187
MSI#2	Tumor	1401604	847841	153	11942	3673	453499	34	33	415	109
MSI#3	Tumor	1477044	1156547	154	10615	1336	149365	28	28	153	92
MSI#4	Tumor	1594617	1186092	128	12557	2790	252820	23	23	235	99
MUTYH#1	Tumor	2290012	570278	82	8732	2237	161069	4	4	142	248
MUTYH#1	Normal	1713073	592137	86	10581	2251	161495	1	1	144	290
MUTYH#2	Tumor	2045145	1275679	151	16567	2581	342119	6	6	308	769
MUTYH#2	Normal	1781825	1371316	184	19248	3154	513681	15	13	550	1076
MUTYH#3	Tumor	2176616	1100083	119	17508	2187	219885	7	6	197	532
POLD1#1	Tumor	3288113	2260525	121	26478	3366	300776	2	2	330	604
POLD1#2	Tumor	2877292	2207696	111	22138	1809	200353	4	4	205	69
POLE#1	Tumor	2883813	2291505	144	21891	2871	426406	49	47	446	115
POLE#1	Normal	2350023	1838529	162	23300	2886	476212	11	11	476	86
WT#1	Tumor	1461481	934129	165	10789	2056	284591	10	8	282	200
WT#2	Tumor	1143725	764774	165	12239	2611	340662	11	11	345	339
WT#3	Tumor	1515289	974796	159	12809	2808	346343	6	6	353	212
WT#4	Tumor	1832678	1320694	151	12930	2348	233688	5	5	215	133
WT#5	Tumor	1408914	960195	151	10381	2093	247685	7	7	225	113
WT#6	Tumor	1846538	1073902	176	17693	2484	389750	9	8	389	657
WT#6	Normal	1645298	967883	163	16896	2806	454818	12	11	368	635
WT#7	Tumor	1595314	873270	161	10372	2231	347887	9	9	348	341
WT#7	Normal	1571865	895592	163	13848	2858	406092	8	7	391	453
HD#1	Normal	1366818	925366	213	9393	4427	1010109	19	17	956	231

*According to the gnomAD v.3 database [https://gnomad.broadinstitute.org/]. SNP – single nucleotide polymorphism.
